# Practicability of serological assays for upscaling COVID-19 laboratory testing in Africa

**DOI:** 10.7189/jogh.11.03038

**Published:** 2021-04-10

**Authors:** Idris Nasir Abdullahi, Anthony Uchenna Emeribe, Olawale Sunday Animasaun, Odunayo RO Ajagbe, Justin Onyebuchi Nwofe, Peter Elisha Ghamba, Chikodi Modesta Umeozuru, Emmanuella Chinenye Asiegbu, Wudi Natasha Tanko, Abdullahi Sani Gadama, Mustapha Bakare

**Affiliations:** 1 Department of Medical Laboratory Science, Faculty of Allied Health Sciences, Ahmadu Bello University, Zaria, Nigeria; 2 Department of Medical Microbiology and Parasitology, Bayero University, Kano, Nigeria; 3 Department of Medical Laboratory Science, Faculty of Allied Medical Sciences, University of Calabar, Calabar, Nigeria; 4 Nigeria Field Epidemiology and Laboratory Training Programme, African Field Epidemiology Network, Abuja, Nigeria; 5 Solina Center for International Research and Development, Abuja, Nigeria; 6 Department of Medical Laboratory Science, Ebonyi State University, Abakaliki, Nigeria; 7 WHO National Polio Reference Laboratory, University of Maiduguri Teaching Hospital, Maiduguri, Nigeria; 8 Department of Medical Laboratory Services, University of Abuja Teaching Hospital, Gwagwalada, Abuja, Nigeria

Several months after the emergence of Severe Acute Respiratory Syndrome Coronavirus 2 (SARS-CoV-2), the etiological agent of the Coronavirus Disease 2019 (COVID-19), the world has experienced unprecedented economic and health uncertainties. As at November 8, 2020, Nucleic acid amplification tests (NAATs) such as the RT-PCR remain the gold standard adopted and recommended by the World Health Organization (WHO) for confirming COVID-19 diagnosis, treatment evaluation and the discharge of cured patients from isolation and/or hospitalization. As at November 10, 2020 there were 1357, 518 RT-PCR confirmed COVID-19 cases and 24 464 associated deaths out of about 12 million tested persons within a population of over 1.3 billion in 47 affected countries and territories in Africa [1,2]. Even though there are efforts by governments of all African nations to scale-up COVID-19 testing capacity and coverage (via house-to-house testing), the number of reported cases is still not an accurate representation of the actual cases in Africa probably. Consequently, many scientists have attributed the relatively low reported COVID-19 cases in Africa to misdiagnosis or underdiagnosis, probably due to low sampling high-risk persons, poor handling of samples, misdiagnosis or/and underdiagnosis, inadequate molecular testing capacity and insufficient manpower required for adequate SARS-CoV-2 molecular testing [3].

Even though it appears that the European and American nations have the highest recorded incidence and worst mortality rates of COVID-19, the relatively low figures reported in Africa (**Table 1**) is not a true reflection of the epidemiology of SARS-CoV-2 infection. This is because many African countries have weak health care systems and hence COVID-19 data may be inaccurate. Some parts of the world are experiencing a second wave of the COVID-19 pandemic, of which there is an increasing need for rapid and reliable SARS-CoV-2 antigen-based and antibody testing, for deployment for either largescale population screening or serodiagnosis purposes [5].

**Table 1:** Top ten COVID-19 most affected africa countries (as at 7:00AM GMT+1, 11 November 2020)*

Country	National population	No. of persons tested	No. (%) with confirmed SARS-CoV-2 infection
South Africa	59 538 871	5 010 350	740 254 (14.8)
Morocco	37 045 375	3 531 400	265 165 (7.5)
Egypt	102 923 202	1 000 000	109 654 (10.9)
Ethiopia	115 824 643	1 534 470	100 327 (6.5)
Nigeria	207 684 434	687 952	64 336 (9.4)
Libya	6 900 014	353 787	70 010 (19.8)
Ghana	31 270 216	551 271	49 302 (8.9)
Kenya	54 130 921	753 959	64.588 (8.6)
Tunisia	11 856 801	387 457	72 993 (18.8)
Cameroon	26 745 630	149 000	22 421 (15.0)

*Source: World COVID-19 daily data [4]. Note: These are ongoing data that could change over time.

Even though SARS-CoV-2 RT-PCR tests provide very accurate results, the availability of their consumables and reagents are usually inadequate in most health care facilities in Africa. Furthermore, RT-PCR tests are quite arduous, costly to operate because they require sophisticated laboratory facilities. Countries with insufficient infrastructure quickly accumulate a backlog of samples for tests and have a long test turnaround time from sample collection (pre-analytical phase) to the availability of tests results (post-analytical phase) [6]. In cognizance of the technical limitations of RT-PCR tests, this article sought to provide supportive data on the performance characteristics and applicability of serology-based assays for upscaling COVID-19 laboratory testing in Africa.

## TECHNICAL LIMITATIONS OF THE STANDARD COVID-19 TESTING PROTOCOL

One of the main technical limitations in the use of RT-PCR tests is false-negative results despite individuals present with clinical and radiologic features highly suggestive of COVID-19. This has been associated with wrong sampling by inadequately sample collectors where SARS-CoV-2 might have been present and replicating in the lower respiratory tracts instead of upper respiratory tracts. Hence, samples often collected will yield accurate test results. This condition will indeed hinder proper evaluation and diagnosis of COVID-19 persons concerned.

**Figure:**
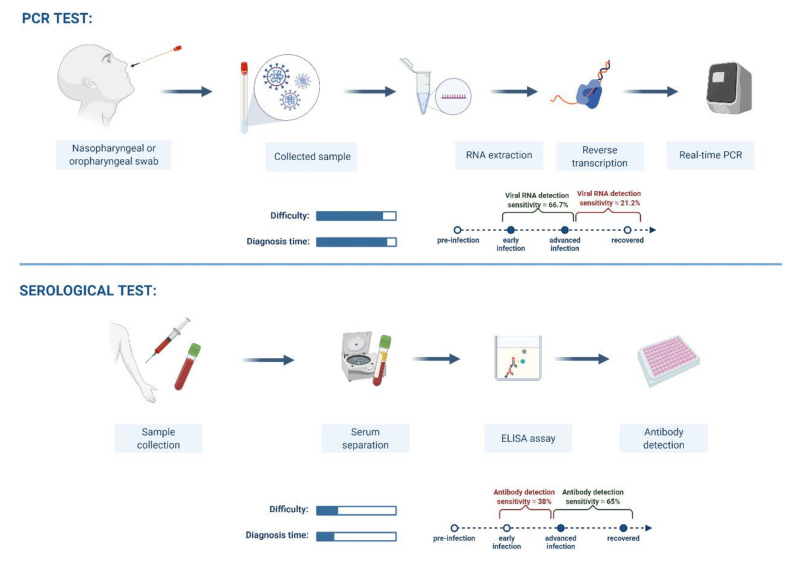
Photo: From the author’s own collection, used with permission.

Other factors include poor sample storage, inadequate transportation conditions, purification and handling, the disintegration of purified COVID-19 RNA, presence of RT-PCR antagonists and viral genetic variants. Besides false-negative reports, RT-PCR has also been reported to be associated false-positive results which can be due to cross-contamination of specimens during sampling, poor pipetting and technical flaws during handling [7-10]. These factors can negatively influence the efficient diagnosis and management of COVID-19 cases.

Till date, there is no categorical explanation on transmission dynamics of SARS-CoV-2 in Sub-Saharan Africa. Significantly, with the reports of the D64G strain of SARS-CoV-2 that could enhance the transmissibility of the pathogen in the population [11]. This mutant alongside others that occurred on genes (N, ORF1ab, E, RdRP) utilized in synthesis RT-PCR primers could also affect the accuracy of the assay and a cause for concern in the reliability of results in locations with records of widespread SARS-CoV-2 mutants. Diagnostically, the N, E and RdRP, ORF1ab are the major targets used in RT-PCR platform. Of note is that mutations in these genes have been reported all over the world. Indeed, the effectiveness and reliability of diagnostic tests are of paramount importance to avoid a large number of false test results if currently used RT-PCR assays are undermined by mutations on SARS-CoV-2 [11-15].

## ACCESSIBILITY AND PRACTICABILITY OF COVID-19 SEROLOGICAL TESTS IN AFRICAN SETTINGS

Aside from the significance of accurate laboratory tests in the diagnosis of COVID-19 index cases, diagnostic testing is also needful for persons who had contact COVID-19 confirmed cases [16]. A certain number of laboratory analytical procedures investigate only contacts that develop symptoms or present with any type of ailment within 14 days after contact. Alternate procedures investigate every contact once detected, irrespective of the presence or absence of symptoms. Researches indicate that a considerable number of infected persons presents no symptoms whatsoever. However, it is worrisome that these asymptomatic persons are nevertheless capable of shedding the virus and infecting susceptible individuals via droplets when they speak, thereby spread infection [17]. Tracing and follow up of every contact of confirmed cases as well as investigating them for SARS-CoV-2 is crucial to contain the pandemic effectively. Serodiagnostics are essential to enable quick surveys to ascertain if and to what magnitude SARS-CoV-2 has spread in a population. This needful in strengthening surveillance systems [16]. This approach has also been useful in monitoring community transmission, identify high-risk individuals and the reliability of preventive strategies.

In the quest to solve the need for sophistication, high-cost running and maintenance, long turn-around-times and an accumulated backlog of samples, some point-of-care molecular tests (such as the GeneXpert equipment) for COVID-19 diagnosis have been developed, calibrated and made accessible for rapid community-based screening. However, these RT-PCR tests have their peculiar limitations, which involve longer cartridges production time and the high demand of these devices by high-income countries have put a strain on the accessibility of the devices, reagents and consumables in low-and middle-income Africa countries [16].

Serological-based tests, which are blood-based have been globally accepted in the evaluation of symptomatic, asymptomatic and recovered cases of COVID-19 by determining the level of exposure of those affected based on the humoral immune response to SARS-CoV-2 infection [16].

Based on applicability, SARS-CoV-2 antigen-based tests have developed and proved to be rapid, easy to perform with a less than 30 minutes turn-around-time when compared with the NAATs [18]. These rapid tests are vital either as alternatives or in supplementing the role of nucleic acid-based tests in the confirmation, management and the signaling of cases to be isolated in order to prevent viral transmission. Furthermore, COVID-19 serological assays are less technical, and the quality of specimen required is relatively lesser than those of RT-PCR tests [18]. Despite these advantages, the applicability of serological-based tests is limited as these kits cannot serve for the screening of COVID-19 due to their low diagnostic performance at the early stage of infection [18].

The early phase of the disease from the time of infection till the period just before the anti-SARS-CoV-2 IgM can be detected in serum (usually from day 3) following infection is the window period. During such period, cases of acute infection are most likely to be missed as the virus remains undetectable within 48 hours from infection [19]. The IgM raised against SARS-CoV-2 continues to rise steadily until the 7th day post-infection when IgG begins to increase while IgM declines [19]. To avoid reporting false negative anti-SARS-CoV-2 IgM or IgG results, these could be resolved by increasing the number of tests done in a week (eg, 3-4 times) to increase the chances of getting true positive antibody test. This helps to narrow the chances of missing a truly infected COVID-19 patients during the “window period” when tested just once. Besides the possible false-negative result during the window period, false-positive can also occur when the SARS-CoV-2 antibodies cross-react with those of other coronaviruses (such as MRES-CoV and SARS-CoV-1) that have high genomic homology with SARS-CoV-2 [20]. For issues of antibodies cross-rection between SARS-CoV-2 and other coronaviruses in 2020, so far there is currently no available report on co-circulation of other coronaviruses with SARS-CoV-2 in sub-Saharan Africa. Hence, the impact of cross-reaction of related viruses on the serological test has little or no effect on the precision of test results. Even though MERS-CoV continues to circulate in the Arabian Peninsula, including countries boarding North Africa, there have not been any confirmed human MERS-CoV infection in Africa, despite the frequent occupational and domestic contact between dromedary animals and humans [21]. However, there is a need for further studies of MERS-CoV at the animal-to-human interface, especially with the recent report on the absence of serological evidence of human MERS-CoV infection in Nigeria [22]. Besides, SARS-CoV-1 has since been exclusively reported in the Asian continent and Europe [23].

Hundreds of serodiagnostic tests devices and kits have so far been developed, and their technical performance characteristics have been made available. Interestingly, the performance characteristics of various serological assays were incredibly encouraging (**Table 2**). Several serological test kits and devices have proved to be reliable for clinical diagnosis due to their excellent diagnostic performance characteristics (ie, accuracy, specificity, sensitivity, negative predictive and positive predictive values) especially during the window period where the SARS-CoV-2 Infection (**Table 2**).

**Table 2:** Global reports of the prospects and limitations of SARS-CoV-2 serological assays

Citation	Study methodology	Key findings	Inferences	Limitation of the study
Lassauniere et al [24]	Evaluated the sensitivity and specificity of 9 commercially serological tests. These included 3 ELISAs and 6 point-POCTs which were validated using serum samples from SARS-CoV-2 PCR-positive patients with a documented first day of disease, archived sera obtained from healthy individuals before the emergence of SARS-CoV-2 in China and sera from patients with acute non- SARS-CoV-2 viral infections	Wantai total antibody ELISA had the best sensitivity (93%) and specificity (100%). However, they reported its potential to cross-react with non-SARS-CoV-2.	The performance of the POCTs generally varied, lower than that of the ELISAs, and discordant with ELISA results.	a. Small sample size. b. The study did not include specimens from mild or SARS-CoV-2 asymptomatic cases c. Samples from previous COVID-19 patients with PCR negative results were not included. d. No serial sampling of each individual to assess the performance of each assay over time p.o.s.
Nicol et al [25]	Evaluation of 2 automated immunoassays (Abbott SARS-CoV-2 CLIA IgG and Euroimmun Anti-SARS-CoV-2 ELISA IgG/IgA assays) and one POCT NGTest® IgG-IgM COVID-19	a. Sensitivity for IgG approximately 80% for CLIA, ELISA, and POCT. b. Sensitivity for IgG detection, >14 d after onset of symptoms, was 100.0% for all assays. c. IgG Specificity was ˃ 98% for CLIA and POCT compared to ELISA (95.8%). d. Specificity was significantly different between IgA ELISA (78.9%) and IgM POCT (95.8%).	Good clinical performance for IgG of the ELISA, CLIA, and POCT at >14 days after symptoms onset. However, it had poor sensitivity during the early days of p.o.s. Therefore, these serological tests can be useful to confirm past COVID-19 and do epidemiologic studies 15 days p.o.s.	a. Subjectivity in the perception of symptoms by patients' symptoms was subjective mainly in elderly patients. b. The study included few patients with asymptomatic infections and positive RT-PCR.
Whitman et al [26]	Evaluation of 10 POCTs and 2 ELISAs anti-SARS-CoV-2 kits/strips on sera of 79 RT-PCR positive and symptomatic individuals; 108 negative controls; and 52 recent samples from individuals with acute non-SARS-CoV-2 respiratory infections.	a. Test specificity ranged from 84.3-100.0% in pre-COVID-19 specimens. b. Specificity was higher when weak LFA bands were considered negative, but this decreased sensitivity. c. IgM detection was more variable than IgG, and detection was highest when IgM and IgG results were combined. c. Agreement between ELISAs and POCTs ranged from 75.7-94.8%. d. No consistent cross-reactivity was observed.	a. Heterogeneous assay performance. b. Re-training of staff needful to reliably read POCTs performance	Assays showed high positive rates within time intervals for more severe disease. Hence, this should be interpreted with caution.
Lin et al [27]	Evaluation of CLIA based on the recombinant N-antigen for diagnosis of SARS-CoV-2 infections and surveillance of antibody changing pattern.	a. The IgG testing was more reliable than IgM in which it identified 65 SARS-CoV-2 infections with sensitivity and specificity of 82.28% and 97.5%, respectively. b. No significant difference in the detection of cases of SARS-CoV-2 infections between the IgM and IgG in patients at a different time of disease onset of disease.	The study provided useful and valuable serological utility of CLIAs of SARS-CoV-2 infections in the community.	COVID-19 patients not classified as symptomatic or asymptomatic. The use of the technique for asymptomatic not fully elucidated
Hoffman et al [28]	Evaluated a commercially available test developed for rapid (within 15 min) detection of SARS CoV-2- IgM and IgG on PCR-confirmed COVID-19 samples cases and negative controls	a. Sensitivities of 69% and 93.1% were recorded for IgM and IgG, respectively, on PCR-positivity b. Specificities of 100% and 99.2% for IgM and IgG were recorded.	a. This indicates that the test is suitable for assessing past SARS-CoV-2 exposure. b. Negative results may be unreliable during the first weeks after infection.	The study is limited because it evaluated only clinical cases and PCR-positives.
Chew et al [29]	Evaluation of Abbott Diagnostics SARS-CoV-2 IgG assay on stored sera from 177 symptomatic COVID-19 positive patients, and 163 non-COVID-19 patients in relation to the time from onset of clinical symptoms to laboratory tests.	The specificity of the assay was 100.0%. However, the sensitivity of the assay varied based on the time from p.o.s, which increased with longer periods since the onset of the clinical symptom (s).	The SARS-CoV-2 IgG protocol has high specificity. But sensitivity was limited in the early stages of the disease which got improved ≥14 days p.o.s.	Limited sensitivity of the IgG assay, especially in the earlier stages of illness. Hence, IgG testing is not suitable for laboratory diagnosis in acute disease but could be considered for retrospective epidemiological purposes.
Yang et al [30]	Measurement of anti- SARS-CoV-2 IgM/IgG and total antibodies by a cyclic enhanced fluorescence assay (CEFA) and a microsphere immunoassay (MIA), respectively. Imprecision, reproducibility, specificity and cross-reactivity (CEFA n = 320, MIA n = 364) were assessed.	The agreement of CEFA and MIA was 90.4%-94.5% in 302 samples. CEFA and MIA detected SARS-CoV-2 antibodies in 26.2% and 26.3%, respectively. Detection rates increased over time reaching 100% after 21 days p.o.s.	Adequately validated CEFA and MIA assays could be considered as reliable for detection of anti-SARS-CoV-2 and showed promise in the clinical evaluation of immune response in hospitalized and convalescent patients, but are not useful for early screening of COVID-19 patients.	a. The study has a relatively small sample size. b. In addition, a small proportion of samples disagreed with CEFA and MIA. separate assays
Ikeda et al [31]	Evaluation of Saliva samples from RT-PCR confirmed COVID-19 (15 asymptomatic and 88 symptomatic). Viral antigen was detected by a rapid antigen immunochromatographic assay.	Viral antigen was detected in 11.7% of all the samples.	The use of rapid antigen tests alone is not recommended for the initial assessment and diagnosis of COVID-19 due to its low sensitivity.	a. The saliva specimens were collected from patients 3 d (median) after confirmed RT-qPCR positive. b. Difficulty in comparing the sensitivity of assays using saliva and other respiratory samples due to variation in viral load in clinical specimens over time.
Kohmer et al [32]	Evaluation of IgG and total antibodies of various structural proteins of SARS-CoV-2 using 4 automated immunoassays (Abbott Architect i2000, Roche cobas, LIAISON®XL platform, and VIRCLIA® automation systems in comparison to two ELISA assays (Euroimmun SARS-CoV-2 IgG and Virotech SARS-CoV-2 IgG ELISA and an in-house developed plaque reduction neutralization test (PRNT).	The N protein-based assays had sensitivity ranged from 66.7 to 77.8% and from 71.1 to 75.6% in the S protein-based assays. Five follow-up samples of three individuals were only detected in either an S and/or N protein-based assay, indicating an individual different immune response to SARS-CoV-2 and the influence of the used assay.	The sensitivity of the examined assays varied. Hence, health care providers need to be careful in selecting commercial serological tests for COVID-19 assessment.	These serological assays could currently be eligible for epidemiological investigations.
Liu et al [33]	Evaluation of 2 ELISA kits based on recombinant SARS-CoV-2 N and S proteins used for the detection IgM, IgG, and their diagnostic performance	a. Out of the 214 patients, 68.2% and 70.1% had detectable N-based IgM and IgG, respectively b. About 77.1% and 74.3% had detectable S-based IgM and IgG, respectively. c. The N-based and S-based ELISAs for IgM and/or IgG positive detection rates were 80.4% and 82.2%, respectively. d. The sensitivity of the S-based ELISA for IgM detection was significantly higher than that of the N-based ELISA. e. An increase in the positive rate for IgM and IgG was recorded with an increase in the number of days p.o.s. However, the positive detection rate for IgM assays dropped after 35 p.o.s.	The positive rate of S-based anti-SARS-CoV-2 ELISAs had appreciable sensitivity, especially for COVID-19 patients after 10 p.o.s.	The use of combined N- and S-based ELISAs are recommended as adjunctive to RT-PCR in the diagnosis of COVID-19.
Theel et al [34]	Evaluation of 4 high throughput serologic tests for anti-SARS-CoV-2 IgG detection, using a panel of serially collected serum samples from 56 COVID-19 confirmed patients and healthy donors.	The Sensitivity of the Abbott, Epitope, Euroimmun, and Ortho-Clinical IgG assays in convalescent serum samples collected ˃14 days p.o.s or after initial RT-PCR positive results were 92.9%, 88.1%, 97.6%, and 98.8%, respectively. Conversely, their specificity and positive predictive values were 99.6%/92.8%, 99.6%/90.6%, 98.0%/71.2% and 99.6%/92.5%, respectively.	The four panels had very good clinical sensitivities. But 14 d p.o.s.	The number of COVID19 confirmed patients was low. The specificity of these assays was not evaluated using samples that were known to be positive for antibodies to the commonly circulating human coronaviruses, to determine cross-reactivity.

p.o.s – post onset of symptom(s), POCT – point-of-care-test, ELISA – enzyme linked immunosorbent assay, CLIA – chemiluminescence immunoassay, PRNT – plaque reduction neutralization test, CEFA – cyclic enhanced fluorescence assay, IgM – immunoglobulin M, IgM – immunoglobulin G, q-RT PCR – quantitative reverse transcriptase polymerase chain reaction

## DRAWBACKS OF SARS-COV-2 SEROLOGICAL TESTS

Sero-diagnostic test kits with high performance characteristics have proved to be mostly reliable especially in areas with high prevalence of the SARS-CoV-2 infection, but they are not recommended for use in isolation for the clinical decision making about SARS-CoV infection in geographical locations with few cases of COVID-19 [35]. This is because there is a significant chance in false-positive test outcomes. On the other hand, false-negative results are also anticipated in high-risk individuals (ie, those with asymptomatic acute phase of the infection, those with other respiratory tract illnesses, those who had contact with confirmed cases) due to low antibody titers which are far below the limited of detection (LOD) of most sero-diagnostic kits and devices. These misleading outcomes can give a false impression of the presence of SARS-CoV-2 infection and misinform public health experts on the appropriate non-pharmaceutical approaches suitable for SARS-CoV-2 prevention and control. The decision to ramp-up testing capacity for public health surveillance and risk assessment by using these sero-diagnostic kits and devices must be guided on the availability of data on the severity and prevalence of the infection in the population of a region [36].

Based on economic reasons, the use of the standard protocol can be restricted to high-risk individuals, especially health care workers, with subsequent follow-up using serodiagnostic medical devices. Previous studies have associated the immunoglobulin level raised against the epitome-binding domain of the SARS-CoV-2 structural proteins with their neutralizing capacity which seems to confer passive immunity in the presence of low viral load [35]. However, there is an inadequate data on the duration of such immunity. Hence, the knowledge gap in understanding the immunological interplay during SARS-CoV-2 reinfections [36,37].

Despite the opinions on passive immunity to avert subsequent SARS-CoV-2 reinfection, the WHO has consistently dismissed this notion based on the paucity of sufficient supporting data on the association between immune status and neutralizing immunoglobulins [38]. In support of the WHO’s stance, several studies have demonstrated instances where infected individuals have high levels of the SARS-CoV-2 nucleic acid in the presence of elevated levels of neutralizing immunoglobulins raised against viral structural proteins (such as the Spike and Nucleocapsid) [39]. Consequently, this reveals that the neutralizing antibodies are poor biomarkers of protective immunity and thus cannot be useful guide (in isolation) to determine the recovery status of SARS-CoV-2 infected individuals. However, the quest to further improve on the accuracy of serological markers for use in evaluating the time of recovery of COVID-19 patients before discharge from the isolation/quarantine or hospital is still ongoing.

## CONCLUSION

The inadequate global cooperation and solidarity during the COVID-19 pandemic have caused many African countries to suffer limited access to molecular diagnostic reagents and consumable for SARS-CoV-2 tests. Furthermore, there is still a paucity of information on the immune response kinetics and dynamics to SARS-CoV-2 Infection, as many reports have not been categorically exhaustive. These have led to hesitation in the use of COVID-19 serological assays, even though these tests are commercially available and scalable. To attain adequate laboratory response required for the adequate containment of the COVID-19 pandemic in Africa, biotechnology development firms, *In-vitro* diagnostics quality assurance laboratories, and regulatory authorities should consider local production, post-production evaluation and standardization of serological tests devices to scale-up COVID-19 testing capacity.
